# First Phytochemical Evidence of Chemotypes for the Seagrass *Zostera noltii*

**DOI:** 10.3390/plants1010027

**Published:** 2012-09-12

**Authors:** Micheline Grignon-Dubois, Bernadette Rezzonico

**Affiliations:** 1 UMR 5805, EPOC, University Bordeaux 1, 351 cours de la Libération, 33405 Talence cedex, France; 2 UMR 5805, EPOC, University Bordeaux 1, 351 cours de la Libération, 33405 Talence cedex, France; Email: b.rezzonico@phyvalbio.u-bordeaux1.fr

**Keywords:** *Zostera noltii*, flavonoid content, apigenin 7-sufate, diosmetin 7-sulfate, chemotype, geographical chemodifferentiation

## Abstract

The variability of the flavonoid content of two populations of *Z. noltii* from different geographical zones, *i.e*., the Bay of Arcachon and the Bay of Cadiz, was evaluated. Samples were collected in spring and autumn at the two sites, and extracts were prepared by maceration in water. The phenolic content was fully characterized using Nuclear Magnetic Resonance (NMR), UV and Liquid Chromatography-Mass Spectrometry (LC-MS), and the concentration of the individual phenolic was determined by quantitative High-Performance Liquid Chromatography with Diode-Array Detection (HPLC-DAD). The two populations show a strong geographical differentiation in their flavonoid content. The samples from Cadiz were dominated by apigenin 7-sulfate, which represents 71% (autumn collection) and 83% (spring collection) of the total flavonoids, whereas the samples from Arcachon were characterized by diosmetin 7-sulfate (85 and 93% of the total flavonoids). Structural elucidation of the individual phenolics was assigned using the complementary information from their spectral evidence. In addition, the results were confirmed by acid hydrolysis of the flavonoid sulfates, and comparison to synthetic standards obtained by sulfation of apigenin, diosmetin and luteolin. The results represent the first experimental evidence of the existence of chemotypes within the species *Z. noltii*.

## 1. Introduction

Seagrasses are a group of about 60 species of rooted vascular plants of terrestrial origin that have successfully returned to the sea. They form the most widespread and productive coastal system in the world, but also one of the most threatened. They grow in large marine meadows, which constitute valuable habitats. Their contribution to the productivity of the oceans has become increasingly recognized over recent decades [1]. The subdivision of the genus *Zostera* (Zosteraceae family) is still under debate, and a discussion continues about dividing or not dividing the genus *Zostera* into two genera, *Zostera* and *Nanozostera* [2]. Nine species belong to this genus, among which *Zostera noltii* constitutes a homogenous group.

Seagrass declines have been reported worldwide either by natural or anthropogenic disturbances [3]. The resilience of seagrass meadows to these events may be strongly mediated by the presence and abundance of secondary metabolite compounds, which could be a factor determining the way the meadow responds to periodic or permanent disturbances. All submersed aquatic angiosperms are secondarily adapted for life in water. A survey of 43 species of seagrass showed that five of the 12 genera, including the genus *Zostera*, had flavonoid sulfates [4]. Flavonoid sulfates have been identified as being of possible taxonomical and ecological significance for seagrasses and other plants of saline habitats, and may play a role in their allelochemical relations [4,5]. Water-soluble compounds from leaves of *Zostera marina* have been reported to exhibit antialgal and antibacterial activities, but the causative substances have not been identified [6,7].

*Z. noltii* Hornem (common name dwarf eelgrass) is an important species of eelgrass occurring along European and North African coasts [1]. *Zostera* beds were severely reduced in Europe due to an outbreak of an epidemic disease in the 1920s. Since then, recovery has been slow and patchy. *Z. noltii* is under increasing threat, with local extinctions recorded for some meadows, and the species is classified as vulnerable and endangered in many parts of Europe. Only a few studies, other than our own [8,9,10], have investigated the concentration of phenolics in *Z. noltii.* Concerning the characterization of the flavonoid profile, only qualitative studies have been reported. Diosmetin- and luteolin 7-sulfates had been previously reported for a Herbarium tissue of *Z. noltii* [4,5]. Diosmetin, diosmetin-7-*O*-glucoside and luteolin-7-*O*-glucoside have been mentioned, but not unambiguously characterized for *Z. noltii* from the Black Sea [11], and luteolin-7-*O*-glucoside for the specimen from Adriatic Sea [12]. However, compound identifications in the latter two were only based on the comparison of the paper electrophoretic mobility or Thin Layer Chromatography (TLC), and the extraction conditions were not adapted to flavonoids and do not meet the current standard. As a result, many of the compounds reported for *Z. noltii*, which were not detected by appropriate phytochemical methods, could represent artifacts.

Until recently, *Zostera* taxonomy was only based on morphology. The development of DNA-based molecular markers has led to an abundant literature on seagrass genetics over the last decade. The existence of geographically distinct populations of *Z. noltii* throughout its biogeographic range has been reported [13,14]. As yet, the factors underlying this geographical genetic variability are poorly understood. Their possible consequences for the phenolic secondary chemistry of *Z. noltii* have not been considered despite the role of these substances as chemical defenses.

Our aim was to fully characterize the flavonoid profile in living tissues of *Z. noltii* and to examine how these compounds vary among seagrass meadows across large geographical areas. This preliminary work reports on *Z. noltii* specimen from two intertidal meadows separated by approximately 1,000 km, namely Arcachon Bay (French Atlantic coast) and Cadiz Bay (Cadiz Gulf, Spain), and the characterization and quantification of their flavonoid content using spectroscopic and chromatographic methods.

## 2. Results and Discussion

**Figure 1:**
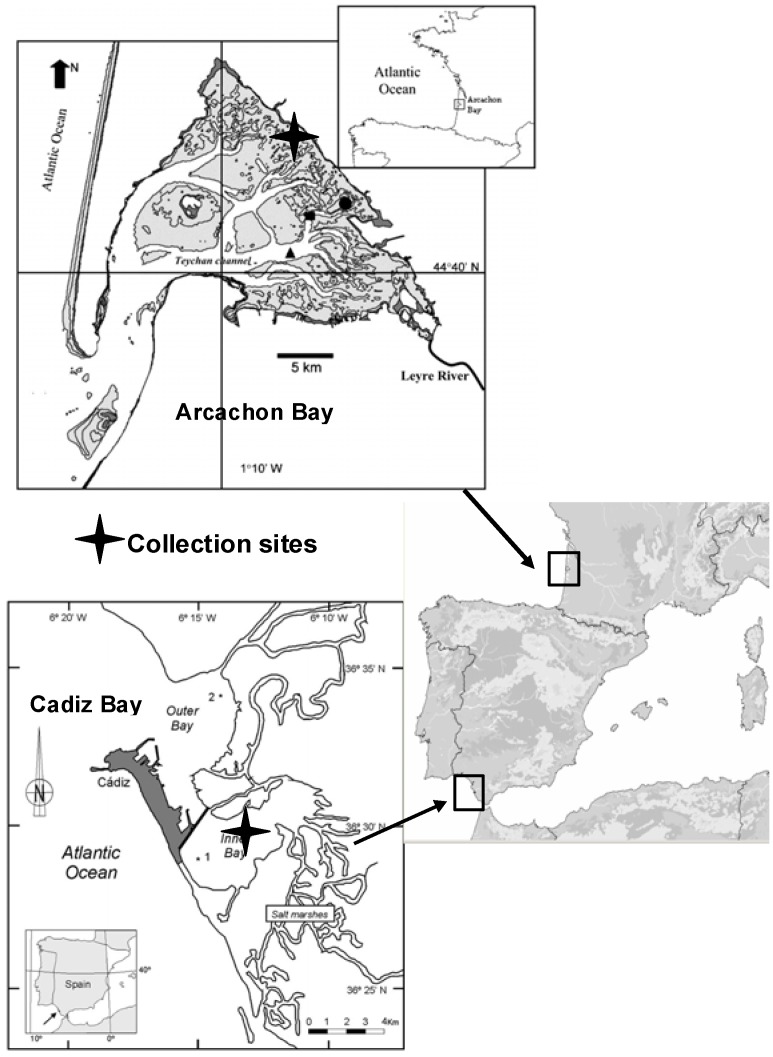
Study sites.

Aqueous extracts of *Z. noltii* leaves were prepared from the sample collected in the Bays of Arcachon and of Cadix (see Figure 1 and Table 1 for details). The crude extracts were analyzed by Nuclear Magnetic Resonance (NMR) and High-Performance Liquid Chromatography with Diode-Array Detection (HPLC-DAD), which both gave a clear understanding of their flavonoid content. ^1^H- and ^13^C-NMR spectra of the crude extracts from Arcachon show a well-defined typical pattern of diosmetin moiety as the major phenolic, whereas crude extracts from Cadiz show the absence of a methoxy group and the typical pattern of an apigenin moiety. In both cases, the shifts observed for ^1^H and ^13^C resonances of ring A are in good agreement with the presence of a sulfate group linked to the C-7 hydroxyl group [15].

**Table 1:** Phenolic amounts in the aqueous crude extracts. Data are expressed as μg (gdw^−1^) of plant material (mean values ± SD of triplicates). Individual compounds are given in order of elution.

Peak number	RT (min)	Compound name	λ_max_ band I (nm)	Cadiz		Arcachon	
				2 Oct. 2007 *	3 June 2008 *	14 Oct. 2007 *	6 June 2008 *
1	9.3	Zosteric acid	283	331 ± 2	298 ± 1	328 ± 2	281 ± 2
2	12.3	Caffeic acid	324	10 ± 1	Tr	339 ± 2	225 ± 2
3	23.6	Luteolin 7-sulfate	348	410 ± 5	256 ± 3	803 ± 8	465 ± 5
4	24.8	Apigenin 7-glucoside	337	98 ± 2	69 ± 2	tr **	tr **
5	26.7	Apigenin 7- sulfate	337	2,410 ± 4	3,600 ± 5	-	-
6	27.3	Diosmetin 7-sulfate	347	319 ± 5	280 ± 5	5,636 ± 12	9,198 ± 9
7	29.5	Luteolin	350	tr **	tr **	25 ± 3	16 ± 2
8	32.6	Apigenin	335	79 ± 3	85 ± 3	-	-
9	33.3	Diosmetin	346	71 ± 3	65 ± 2	159 ± 3	216 ± 5
		**Total flavonoids**		3,378	4,355	6,623	9,895

* sampling dates; ** tr: traces.

**Figure 2:**
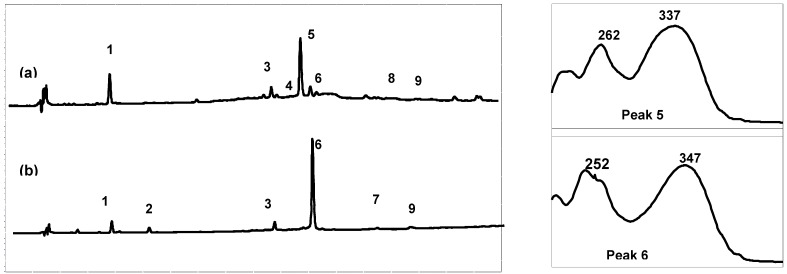
Comparison of the High-Performance Liquid Chromatography with Diode-Array Detection (HPLC-DAD) profile (280 nm) of the crude extracts and online UV spectra of the major products: (**a**) Cadiz, (**b**) Arcachon. 1: zosteric acid; 2: caffeic acid; 3: luteolin 7-sulfate; 4: apigenin 7-glucoside; 5: apigenin 7-sulfate; 6: diosmetin 7-sulfate; 7: luteolin; 8: apigenin; 9: diosmetin.

**Figure 3:**
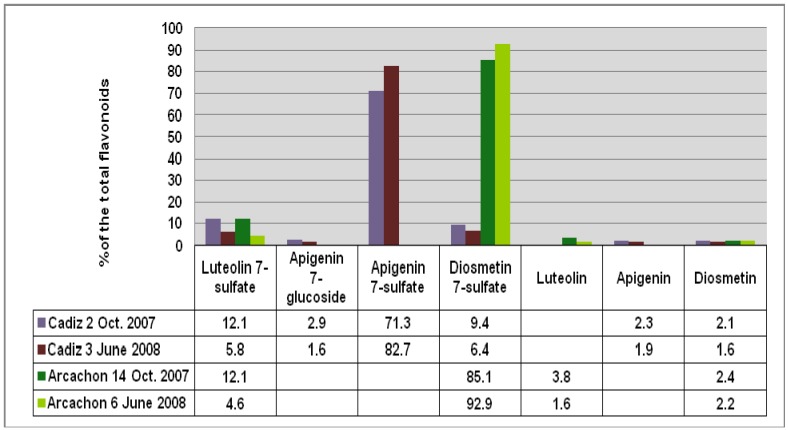
Comparison of the flavonoid content at each site given as % of the total flavonoids detected (FT).

High performance liquid chromatography (HPLC) combined with diode array detection (DAD) was used for both qualitative and quantitative analyses of the extract composition (Table 1). The results show that the Arcachon samples contain higher amounts of flavonoids than the Cadiz samples (6,623 and 9,895 *versus* 3,378 and 4,355 μg/g, respectively). As expected on the basis of the NMR data, the HPLC flavonoid profiles of the four extracts were largely dominated by a single product, which was eluted at 26.3 min (peak 5, on-line λ_max_, 337 nm; Cadiz) and 27.3 min (peak 6, on-line λ_max_, 347 nm; Arcachon) (Figure 2), respectively. Apigenin 7-sulfate accounted for 2,410 μg/g (Cadiz, autumn 2007 collection) and 3,600 μg/g (Cadiz, spring 2008 collection), representing 71 and 83%, respectively, of the total flavonoids (TF) detected (Figure 3). In contrast, apigenin 7-sulfate was not found in the samples from Arcachon, which were dominated by diosmetin 7-sulfate with 5,636 μg/g (autumn 2007, 85% of TF), and 9,198 μg/g (spring 2008, 93% of TF). Diosmetin 7-sulfate is also found in Cadiz, but as a minor product accounting for only 410 and 256 μg/g, which represents 9 and 6% of the TF (Figure 3). In addition, small amounts of apigenin 7-*O*-glucoside (Peak 4, on-line λ_max_, 335 nm) were detected in Cadiz, but not in Arcachon. Luteolin 7-sulfate was found as a minor product at the two locations (Peak 3). The comparison of the HPLC-DAD profiles (Figure 2), and the flavonoid composition expressed as a percentage of the TF detected at each site (Figure 3) clearly shows the dramatic geographical chemodifferentiation between the two study sites.

All the UV absorptions are in agreement with the literature [4,16]. The structural assignments were supported by LC-ESI-MS analysis in positive mode. In particular, the mass spectra clearly show the [M+1] molecular peak for all the flavonoids detected, and the characteristic ion peak at [M+1-80] for sulfated flavonoids or [M+1-162] for gluco-flavonoids. The linkage of the sulfate moiety to the 7-position was established from the UV [5,16] and NMR data [15].

Our results were confirmed by acid hydrolysis of the crude extracts, which led to diosmetin (Arcachon) and apigenin (Cadiz) as the major product (comparison with standards). In addition, authentic samples of the 7-sulfated flavonoids were synthesized by sulfation of luteolin, apigenin and diosmetin with tetrabutylammonium hydrogen sulfate [15]. Comparison of the NMR, MS and UV spectra and HPLC retention time allows the unambiguous identification of the sulfated flavonoid content of *Z. noltii* from Arcachon and Cadiz.

Zosteric acid was also found as a minor compound at the two study sites (see Table 1), and caffeic acid in the samples from Arcachon. Zosteric acid is a highly hydrophilic sulfated coumaric acid, which is known to prevent settlement of some marine bacteria, algae, barnacles and tubeworms at low concentration [9].

The sulfate component is believed to represent a marine adaptation [17]. Sulfate is the third highest ion in concentration in seawater and hydrogen sulfide is commonly found in anoxic marine sediment. Harborne evoked the possibility of flavonoid sulfates having a dynamic function in salt uptake and metabolism [17]. Nissen and Bessen [18] found that 50% of the radiolabeled sulfate fed to *Zostera marina* was recovered in the phenolic flavonoid fraction. Taxonomic and ecological implications were evoked by McMillan *et al*. [4]. The role of sulfated flavonoids in seagrasses remains unclear and has yet to be documented. Nevertheless, there is increasing evidence that these hydrophilic substances have a role to play in the physiological survival of seagrasses in the marine environment. Luteolin 7-*O*-D-glucopyranosyl-2-sulfate isolated from the tropical seagrass *Thalassium testidinum* has been shown to chemically defend the seagrass against zoosporic fungi [19]. We have recently shown that aqueous extract of *Z. noltii* from the Bay of Arcachon and the Thau lagoon significantly inhibits the growth of the Harmful Algal Bloom (HAB) *Alexandrium catenella*. The highest concentrations of phenolics were found to correspond to the lowest EC_50_ values, suggesting that these metabolites might be responsible for the observed algicidal activity [20].

This is the first time sulfated flavonoids have been quantified in *Zostera* species. Apigenin 7-sulfate has never been reported for *Z. noltii* before. Long-term monitoring of the phenolic content in monthly-collected fresh leaves of *Z. noltii* from the Arcachon lagoon is now in progress in our laboratory. From the results acquired since 2007, it appears that diosmetin 7-sulfate was the only major flavonoid sulfate whatever the season, while apigenin 7-sulfate has never been detected [21]. Based on these data, our results show that *Z. noltii* grown in Cadiz Bay is chemically distinct from specimens grown in Arcachon Bay. 

Only a few chemotaxonomical studies have been reported for some seagrasses. They were generally conducted at the intergeneric or interspecific level [22]. To the best of our knowledge, the only study at the specific level was reported for *Halophila ovalis* subspecies populations of the Pacific, Indian Ocean and Australia, which differ in the occurrence of sulfated flavonoids on the basis of morphological variations and geographical distribution [23,24].

**Figure 4:**
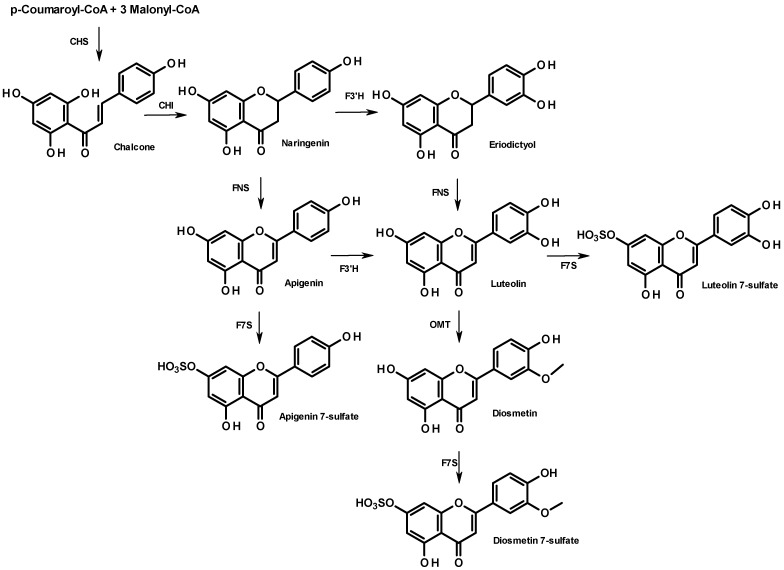
Biosynthesis of apigenin- and diosmetin-7-sulfate in the flavonoid pathway. Enzyme names are abbreviated as follows: chalcone isomerase (CHI), chalcone synthase (CHS), flavanone 3'-hydroxylase (F3'H), flavone synthase (NFS); flavone *O*-methyltransferase (OMT), flavonoid 7-sulfotransferase (F7S).

From a biosynthetic point of view, flavonoid compounds result from the stepwise condensation of three molecules of malonyl CoA and one molecule of 4-coumaroyl CoA followed by a stereospecific cyclization leading to a flavanone [25]. All flavonoids are derived from a limited number of flavanone intermediates, which serve as substrates for a variety of enzyme activities, enabling the generation of diversity in flavonoid structures. The biosynthesis of the 7-sulfate of apigenin, diosmetin, and luteolin is summarized in Figure 4. They all share the same metabolic flavanone precursor, naringenin, but the subsequent steps differ. While naringenin leads directly to apigenin (FNS step), followed by apigenin 7-sulfate (F7S step), diosmetin 7-sulfate can originate from two distinct pathways: through apigenin or through eriodictyol, which both involve a flavonoid 3'-hydroxylase (F3'H). The lack of diosmetin 7-sulfate in the sample from Cadiz suggests a poor expression of the gene encoding F3'H. Further research will be needed to elucidate the different metabolisms of these two populations. In particular, it would be of interest to perform a cross phytochemical/phylogenetic analysis of *Z. noltii* to correlate the phenolic fingerprint and the amino acid sequences of genes encoding the flavonoid pathway.

This is the first report of quantitative data on the individual flavonoids in *Z. noltii* and the first report of the existence of chemotypes within the Zosteraceae family. This work reveals unknown features about the chemical plasticity and patterns of the phenolic composition in *Z. noltii* and shows the need for tandem phytochemical and genetic studies of this species throughout its biogeographic range. Understanding the underlying causes of the geographic variation of the *Z. noltii* sulfated flavonoid content and its possible link with ecological factors appears crucial to elucidating the functioning of *Z. noltii* communities, and for monitoring and managing *Zostera* beds. Fingerprinting of specimens collected at twelve localities throughout the Atlantic and Mediterranean is now in progress.

## 3. Experimental Section

### 3.1. General Methods

The solvents used were all HPLC-grade. Standards were purchased from Extrasynthèse (Genay, France), and all the chemical reagents used were from Aldrich Chemical Company. ^1^H-, ^13^C-NMR and 2D-NMR spectra were recorded on an AVANCE 300 MHz instrument (Bruker) in DMSO-d6 (Euriso-Top, Gif-Sur-Yvette). Chemical shifts are expressed in δ (ppm) values relative to tetramethylsilane (TMS) as an internal reference. Coupling constants are reported in hertz (Hz). ^13^C-NMR assignments were made by 2D HSQC and HMBC experiments. High performance liquid chromatography (HPLC) combined with diode array detection (DAD) was performed on a Thermo Electron liquid chromatography system. LC-MS was performed using a HP1100 (Hewlett-Packard) equipped with an Agilent MSD 1946B simple quad mass spectrometer and an HP Chemstation software.

### 3.2. Study Sites and Plant Collection

The two study sites are intertidal monospecific *Z. noltii* meadows. Both are exposed to long periods of desiccation and to rapid variations and extreme values of temperature, light intensities and salinity.

The Bay of Cadiz (SW Spain; 36°23'-36°37'N, 6°09'-6°21'W) is located in the Atlantic Ocean, close to the Mediterranean Sea and to Northern Africa (Figure 1). The bay is subdivided into two basins, a shallower basin (inner bay), with a maximum depth of 11 m, and a deeper basin (outer bay) with a maximum depth of 17 m. In Cadiz Bay, *Z. noltii* beds are extensive and colonize the major part of the exposed intertidal area.

The Bay of Arcachon (Figure 1) is a 155 km^2^ mesotidal system located on the south-western French Atlantic coast (44°40'N, 1°10'W). It opens to the ocean via a narrow channel. Approximately 130 × 106 m^3^ (neap tide) and 400 × 106 m^3^ (spring tide) of water are exchanged between the lagoon and the ocean during one tidal cycle. The tidal amplitude ranges from 1.10 m on neap tides to 4.95 m on spring tides, and the local mean sea level (MSL) is +2.20 m, relative to French marine 0. Rivers and streams, mainly located in the northern and eastern parts of the Bay, provide a freshwater inflow of approximately 14,000 m^3^/d. *Z. noltii* beds are extensive in the Arcachon Bay and colonize the major part of the exposed intertidal area between −1.9 m and +0.8 m relative to the local Mean Sea Level. The period of meadow emersion during low tide is long (10-14 h). The sampling station was located in Andernos (inner part of the Bay).

Thirty shoots of *Z. noltii* Hornem. (Zosteraceae) were sampled in the growing season in 2007 and 2008 from intertidal monospecific meadows in Andernos (Arcachon Bay, 14 October 2007 and 6 June 2008) and El Bajo de la Cabezuela (Cadiz Bay, 2 October 2007 and 3 June 2008). Plants were gathered carefully to keep belowground parts intact and transported to the laboratory. After collection, the samples were thoroughly rinsed in seawater, and then quickly washed in freshwater to remove sand and salt. The collected material was handpicked to remove associated debris, and leaves were separated from rhizomes. Then, the plant material was air-dried at room temperature to a constant weight. The moisture content of the dried material was <1%. Leaves were manually ground using a mortar and pestle immediately before extraction.

### 3.3. Extraction and Flavonoid Content Determination

The pulverized air-dried leaf material (10 g) was extracted by maceration in water for 24 h at room temperature. The process was repeated twice, and then the extracts were pooled together and freeze-dried yielding an amorphous powder. Extraction yields were: 27.6 and 25.4%, respectively for the Cadiz samples; 30 and 25.1%, respectively for the Arcachon samples (given as % of the seagrass dry weight).

### 3.4. Quantification of the Phenolic Content

Separation and quantification of phenolics in the crude extracts were performed using high-performance liquid chromatography, consisting of a liquid chromatography system (Thermo Electron) equipped with a SCM 1000 solvent degasser, a thermostatically controlled column apartment, an AS 3000 autosampler with a 100 μL loop, a PDA UV6000LP detector and a Chromquest Chromatography Workstation. Separations were carried out at 40 °C on a Hypersil GOLD C8 column (Thermo Finnigan), 175 A° pore size, 5 μm particle size, 250 × 4.6 mm i.d. column. The analytes were eluted at a flow rate of 1 mL/min using the binary gradient 0.1% (v/v) TFA in water (A) and methanol (B). The following linear gradient was used: zero min, 1% B; 60 min, 99% B. Run time was 60 min, stop time was 60 min, post time was 10 min. UV spectra were collected over the range of 220-440 nm, and the chromatograms were recorded at 270, 328 and 350 nm with a resolution of 1 nm and no smoothing. In addition, the data were processed to create a chromatogram, in which each chromatographic peak represents the absorbance of the eluting substance at its l_max_ (max-plot chromatogram). The injection volume was 20 μL. The data were integrated using the Chromquest automated software system. Stock solutions of the dried extracts were prepared in DMSO at a concentration of 0.5 mg/mL. All solutions were filtered prior to analysis through a 0.2 μm syringe filter and injected three times into the HPLC. Chromatographic peaks were checked for peak purity and identification was achieved by comparing retention times and UV spectra with those of standards and authentic samples obtained by sulfation of apigenin, diosmetin and luteolin.

Quantitative determinations of flavonoids were carried out by peak area measurements at 350 nm, using an external calibration curve of diosmetin dissolved in DMSO. The curve was established on six data points, covering the concentration range 0.0619-0.00619 mg/mL. Linear regression on the HPLC analyses gave R^2^ values of 0.9994.

Quantitative determinations of zosteric acid were carried out by peak area measurements at 280 nm, using a calibration curve of coumaric acid [9]. The linear regression coefficient was 0.9998 (six points).

Quantitative determinations of caffeic acid were carried out by peak area measurements at 328 nm, using a calibration curve of caffeic acid at the same wavelength (0.9996, six points).

The data presented in Table 1 are the average from three experiments, calculated using the following equation:

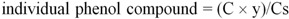
(1)
where C is the concentration of the tested phenolic compound (mg/mL) in the analyzed extract, calculated from peak areas and linear regression; y is the extraction yield; Cs is the concentration of the sample (mg/mL), diluted in DMSO/deionised water 4:1 (v/v).

Data are expressed in micrograms per gram of dry matter of *Z. noltii* (μg/gdw; mean ± standard deviation (SD) of three determinations).

### 3.5. LC-MS Analyses

HPLC-PDA-ESI/MS analyses were performed using a HP1100 (Hewlett-Packard) equipped with an Agilent MSD 1946B simple quad mass spectrometer and a HP Chemstation software. Positive mode ESI spectra of the column eluate were recorded in the range of *m/z* 100–1,000 a.m.u. Absorbance was measured at 280 and 320 nm. Compounds were separated using an MN Nucleodur C18 column (Macherey-Nagel, Germany) measuring 125 mm × 2 mm i.d, 3 µm particle size. The analytes were eluted at a flow rate of 0.3 mL/min using the binary gradient (v/v) formic acid in water (1%, pH = 2.55, A) and methanol (B). The following linear gradient was used: fifteen percent B to 100% B (15 min). Separation of the analytes was carried out at 50 °C. The injection volume was 2 µL. For mass spectrometric analysis, compounds were detected using the following conditions: nebulising gas pressure, 60 psi; drying gas flow rate, 12 L/min; temperature, 350 °C; capillary voltage, 4,000 V; temperature source, 350 °C. Data were acquired in full scan mode (*m*/*z* 100–1,000) at a fragmentor voltage of 70 V.

### 3.6. Acid Hydrolysis of the Crude Extracts

One hundred milligram samples of crude extract from Arcachon and Cadiz were separately dissolved in 100 mL of methanol and stirred with 5 mL of TFA at room temperature until total disappearance of the sulfated flavonoids as monitored by HPLC. After evaporation of methanol under *vacuum*, the reaction mixture was partitioned between *n-*butanol and water. Addition of BaCl_2_ to the aqueous layer gave a white precipitate of BaSO_4_. The butanolic fraction was evaporated to dryness, then analyzed by HPLC, UV and NMR, and compared with authentic samples of apigenin, diosmetin and luteolin. Results showed the large predominance of apigenin in the case of Cadiz and diosmetin in the case of Arcachon. Small amounts of luteolin were also found in the hydrolysis mixtures, which confirms the presence of luteolin 7-sulfate at the two locations.

### 3.7. Synthesis of the 7-Sulfated Flavonoid Standards

We used the dicyclohexylcarbodiimide-mediated selective 7-sulfation of flavones with tetrabutylammonium hydrogen sulfate as described by Barron and Ibrahim for apigenin and luteolin [15]. Spectral data for the resulting sulfated products were identical to those described in the literature for apigenin 7-sulfate, and luteolin 7-sulfate (NMR, UV, MS) [15,16], and diosmetin 7-sulfate (UV) [5].

*Diosmetin 7-sulfate*: d_H_ (300 MHz, DMSO-*d_6_*) 3.86 (3H, s, OCH_3_), 6.53 (1H, d, *J* 1.67, H-6), 6.84 (1H, s, H-3), 7.04 (1H, d, *J* 1.43, H-8), 7.09 (1H, d, *J* 8.59, H-5'), 7.49 (1H, d, *J* 2.14, H-2'), 7.58 (1H, dd, *J* 8.58 and 2.14, H-6'); d_C_ (75.47 MHz, DMSO-*d_6_*) 54.13 (OCH_3_), 96.13, 100.6, 102.06, 110.65, 111.28, 117.27 (6CH), 121.18, 145.22, 149.7, 154.76, 157.91, 158.89, 162.46 (7C), 180.49 (CO); LC-ESI-MS *m/z* 381 (M+1), 301 (M+1-80, 100%). UV λ_max_ (band I): 343 nm (MeOH); HPLC-DAD online 347 nm (RT 27.3 min).

## 4. Conclusions

The present study is the first effort to compare and fully characterize the sulfated flavonoid profile in two populations of the seagrass *Z. noltii*. Results show that each population is largely dominated by a specific flavonoid: apigenin 7-sulfate in Cadiz and diosmetin 7-sulfate in Arcachon. This well-marked chemodifferenciation fits well with the recent genetic data reported in the literature for the Cadiz group. The results represent the first experimental evidence of the existence of chemotypes within the species *Z. noltii*. They demonstrate the need to correlate data obtained with DNA-based molecular markers and the secondary chemistry in addition to morphology.
